# Biofilm Formation and Antimicrobial Susceptibility of *Staphylococcus epidermidis* Strains from a Hospital Environment 

**DOI:** 10.3390/ijerph110504619

**Published:** 2014-04-25

**Authors:** Robert D. Wojtyczka, Kamila Orlewska, Małgorzata Kępa, Danuta Idzik, Arkadiusz Dziedzic, Tomasz Mularz, Michał Krawczyk, Maria Miklasińska, Tomasz J. Wąsik

**Affiliations:** 1 Department and Institute of Microbiology and Virology, School of Pharmacy with the Division of Laboratory Medicine, Medical University of Silesia, ul. Jagiellońska 4, 41-200 Sosnowiec, Poland; E-Mails: rwojtyczka@sum.edu.pl (R.D.W.); kamila_orlewska@o2.pl (K.O.); mkepa@sum.edu.pl (M.K.); didzik@sum.edu.pl (D.I.); tomekmularz@aol.com (T.M.); michalk1988@interia.pl (M.K.); mariii89@o2.pl (M.M.); 2 Department of Conservative Dentistry with Endodontics, School of Medicine with the Division of Dentistry, Medical University of Silesia, Pl. Akademicki 17, 41-902 Bytom, Poland; E-Mail: adziedzic @sum.edu.pl

**Keywords:** biofilm, nosocomial infections, *icaADBC* operon, *Staphylococcus epidermidis*

## Abstract

The hospital environment microflora comprise a wide variety of microorganisms which are more or less pathogenic and where staphylococci are one of the most common types. The aim of the presented study was to evaluate the prevalence of the biofilm forming coagulase-negative staphylococci (CoNS) in a hospital environment as a risk factor for nosocomial infections. Among 122 isolated and tested strains of CoNS the most frequent were: *S. epidermidis*—32 strains, *S. haemolyticus*—31 strains, *S. capitis* subsp. * capitis*—21 strains, *S. hominis*—11 strains*, S. cohnii* subsp*. cohnii*—nine strains. In case of CoNS, the main molecule responsible for intercellular adhesion is a polysaccharide intercellular adhesin (PIA), encoded on the *ica* gene operon. The analysis revealed the presence of the *icaADBC* operon genes in 46.88% of *S. epidermidis* isolates. *IcaA* and *icaD* were present in 34.38% and 28.13% of strains respectively while *IcaC* gene was present in 37.50% of strains. *IcaB* gene was found in 21.88% of *S. epidermidis* strains. In 15 (63%) strains all *icaADBC* operon genes were observed. The assessment of antibacterial drugs susceptibility demonstrated that analyzed CoNS strains were highly resistant to macrolides and lincosamides and more sensitive to rifampicin and linezolid. Our data indicates that the hospital environment can be colonized by biofilm forming coagulase-negative staphylococci and transmission of these strains can cause an increased risk of serious nosocomial infections.

## 1. Introduction

According to The European Centre for Disease Prevention nosocomial infection are identified in approximately three million people in the European Union each year and about 50,000 of them are fatal [1]. These infections can also affect medical personnel, patient’s visitors and hospital support staff [2]. Approximately 60%–70% of nosocomial infections are associated with the use various types of medical-devices with surfaces contaminated with pathogenic bacteria [3] and what is more, the contaminated hands of medical personnel are also considered as one of the pathways of nosocomial infection spread. It has been shown that proper use of disinfectants can significantly reduce microorganism content and thus reduce the risk of hospital associated nosocomial infections by more than 40% [4]. Due to the reduced susceptibility of biofilm forming microorganisms to antibiotics [5,6] and some disinfectants [7] commonly used skin disinfection techniques seems to be inefficient. Therefore to reduce the risk of infection associated with these microorganisms it is important to introduce more rigorous disinfection procedures to remove biofilm strains from the general hospital environment.

Many microorganisms in the natural environment are organized in biofilm structures [8]. Biofilms can be defined as multicellular communities of bacteria, immobilized by an extracellular polymeric matrix produced by the bacteria, which can be attached to various biotic and abiotic surfaces [9,10]. This three-dimensional biofilm structure is made up in 85% by the extracellular matrix which comprises polysaccharides, proteins, enzymes, DNA, bacterial glycolipids, water, and in 15% by aggregates of microorganism cells [8]. Biofilm development depends on many physical, chemical and biological factors [3]. In staphylococci, the main molecule responsible for intercellular adhesion is a polysaccharide intercellular adhesin (PIA), also known as a poly-N-acetylglucosamine (PNAG) [11]. It is a partially deacylated polymer of β-1,6-N-acetylglucosamine, which with the other polymers such as teichoic acids and proteins can form a major part of the extracellular matrix. Recently, PIA homologs were identified in many pathogens with biofilm formation ability, what points out that the three-dimensional matrix formation plays a crucial role in bacterial virulence in biofilm-associated infections [12,13,14].

PIA biosynthesis is carried out by the proteins encoded by the *ica* gene operon: N-acetylglucosamine transferase (*icaA* and *icaD*), PIA deacylase (*icaB*), PIA exporter (*icaC*) and the regulatory gene (*icaR*) [15,16]. *Ica* locus expression is regulated by a variety of environmental factors and internal regulatory proteins. Biosynthesis and deacetylation of PIA are recognized as crucial virulence factors in *Staphylococcus epidermidis-* associated infections [15,17,18]. 

Biofilms protects microorganisms, such as coagulase-negative staphylococci (CoNS), against both antibiotics used to treat infections and host immune system responses. Medical implants contaminated by biofilm-forming bacteria may lead to the development of inflammatory foci where implant removal is frequently the only effective treatment of such infections [19,20,21]. Since *Staphylococci* are part of the resident microbiological flora of the skin the presence of the biofilm-forming strains among them may be associated with an increased risk of transmission of virulent biofilm-forming strains in the hospital environment.

CoNS colonization in humans occurs as early as at birth and many strains inhabit the skin and mucous membranes till death [9]. Among many coagulase-negative staphylococci *Staphylococcus epidermidis* is the most frequently isolated species and accounts for more than 90% of the aerobic flora [22]. Although, this very common species of the cutaneous microflora is believed to be generally innocuous in nature, the last 20 years have pointed at *S. epidermidis* as a very frequent cause of hospital-acquired infections [23]. Therefore, we made an effort to evaluate the prevalence of biofilm-forming *S. epidermidis* and other CoNS strains present in the hospital environment by both phenotypic and molecular methods. Acquired data, together with the antimicrobial susceptibility profile of the isolated strains, may provide important epidemiological information which can be implemented in hospital infection prevention and control plans.

## 2. Experimental Section

A total of 122 coagulase-negative staphylococci strains isolated from a hospital environment were included to the presented study. The samples were collected from air and surfaces in the hospital. The sedimentation method was used for the air sample collection, where Petri dishes containing nutrient growth medium were exposed to the environment for one hour [24].

Standard Replicate Organism Detection and Counting (RODAC) contact plates (BTL, Łódź, Poland) contained neutralizing agents which inactivated any residual disinfectants were used for collection of bacteria from surfaces. The convex agar meniscus allowed direct application to the tested surfaces e.g. walls, floors, medical equipment, equipment for hygiene control [25].

Routine microbiological methods with a semi-automatic MICRONAUT identification system (Merlin-Virotech, Bornheim-Hersel, Germany) were used for bacterial species identification. Samples were stored for future analysis in TBE medium with 20% glycerol, at −86 °C.

### 2.1. Analysis of Biofilm Production by the Congo Red Agar (CRA).

Phenotypic characterization of biofilm production was performed by culture of the CoNS isolates on CRA plates as previously described by Freeman *et al.* [26]. A specific brain-heart infusion broth (BHI) medium supplemented with 5% sucrose and Congo Red was prepared. The medium was comprised BHI (37 g/L), sucrose (50 g/L), No. 1 agar (10 g/L) and Congo Red stain (0.8 g/L). Plates were inoculated and incubated in aerobic environment for 24 h at 37 °C. Under such condition, biofilm producers form black crusty colonies on CRA, whereas non-producers form red colonies. A darkening of the colonies with the absence of a dry crystalline colonial morphology indicated an intermediate result [27].

### 2.2. Microtiter Plate Assay (TCP)

To evaluate the biofilm formation we performed the modified microtiter plate assay described by Christensen *et al.* [27]. Bacteria were suspended in Muller-Hinton Broth (MHB-BTL, Łodź, Poland) in density equivalent to 0.5 McFarland standard and 100 μL from each bacterial suspension was inoculated onto 96-well tissue microculture plates. The plates were incubated at 37 °C for 24 h in a normal atmosphere. To remove the free floating planktonic bacteria the medium was removed and the wells were washed 3 times with phosphate saline buffer (PBS, pH = 7.2). Then 150 μL of 1% crystal violet (Sigma) was added to each well and incubated for 30 min at room temperature. The dye was removed, by 4× wash with sterile deionized water. The samples were incubated with 200 μL of 95% isopropanol in 1 M HCl for 5 min. Finally, 100 μL of colored isopropanol from each well was transferred to a fresh microtiter plate. The optical density (OD) of suspension was measured at wave length of 490 nm with a Multitec SX microplate reader.

The negative control comprised all reagents but without bacterial inoculums. According to Christensen *et al.* [27], the samples with the OD > 0.11 should be considered as positive. The assay was performed in triplicates. Mean A_490_ ± SD values were calculated.

In the present study, bacterial strains were considered non-adherent when the OD was equal or lower than 0.11; weakly adherent when the OD was higher than 0.11 or equal or lower than 0.17 and strongly adherent when the OD was higher than 0.17.

### 2.3. Detection of icaADBC Genes S. epidermidis Strains

Bacterial DNA was isolated using Genomic DNA Mini Kit (BLIRT SA, Gdańsk, Poland). Briefly, all isolates stored at −86 °C were thawed and expanded *in vitro* on blood agar plates and checked for strain purity prior to the DNA isolation. In the next step 3–4 bacterial colonies were suspended in 100 μL TRIS buffer with 10 μL of lysostaphine (1 mg/mL; BLIRT SA, Gdańsk, Poland) and incubated at 37 °C for 30 min. The mix was treated with proteinase K and LT Buffer at 37 °C for overnight than incubated at 75 °C for 5 min. The DNA samples were purified according to the protocol with the use of ethanol and wash buffer supplied in the kit, and finally diluted to 200 μL with TRIS buffer. Purified DNA samples were stored at −20 °C for future analysis.

A standard PCR technique was used for *icaA, icaD, icaB* and *icaC* genes detection in the *S. epidermidis* strains as earlier described by Ziebuhr *et al.* [28] and de Silva *et al.* [29]. The primer sequences for *icaA* (f) were 5’-GAC CTC GAA GTC AAT AGA GGT-3’ and *icaA* (r) 5’-CCC AGT ATA ACG TTG GAT ACC-3’; *icaD* (f) were 5’-AGG CAA TAT CCA ACG GTA A-3’ and *icaD* (r) 5’-GTC ACG ACC TTT CTT ATA TT-3’ (reverse); *icaB* (f) 5’-ATA AAC TTG AAT TAG TGT ATT-3’ and *icaB* (r) 5’-ATA TAT AAA ACT CTC TTA ACA-3’ *icaC* (f) 5’-AGG CAA TAT CCA ACG GTA A-3’ and *icaC* (r) 5’-GTC ACG ACC TTT CTT ATA TT-3’.

The PCR reactions were performed using 10× PCR RED master mix kit (BLIRT SA). The PCR mix contained 2 μL of PCR red mix, 0.2 μL of each primer, 16.6 μL of PCR water and an average of 75 ng of DNA per 20 μL of reaction mix. The PCR reaction was performed in 30 cycles with 30 s of denaturation at 95 °C and 3 min of elongation at 72 °C for all reactions, and with annealing for 1 min at 60 °C (*icaA*), 59 °C (*icaD*, *icaB*), 45 °C (*icaC*). The PCR was performed using a MJ Mini Personal thermal cycler (Bio-Rad, Hercules, CA, USA). PCR products were electrophoresed in 1.5% agarose gel containing 0.5 μg/mL ethidium bromide. The bands were visualized by ultraviolet illumination by an UVP Bioimaging System (UVP Inc., Upland, CA, USA) and checked for size against molecular weight markers using 1 Kb HyperLadder IV (BLIRT SA)

### 2.4. Antimicrobial Susceptibility Testing

The antimicrobial susceptibility to cefoxitin (FOX), erythromycin (E), clindamycin (DA), tetracycline (T), chloramphenicol (C), ciprofloxacin (CIP), gentamicin (CN), rifampicin (RIF), linezolid (LIN) and trimethoprim/sulphamethoxazole (SXT) was tested by disk-diffusion method and interpreted according EUCAST guidelines [30]. Commercial antibiotic discs (EMAPOL, Gdańsk, Poland) and Mueller-Hinton agar medium (BTL, Łódź, Poland) were used in this tests. 

### 2.5. Statistical Analysis

The two-sided Fisher’s exact tests was use to assess the relationship between the capacity of the biofilm formation and drug resistance. The Pearson chi-square was used to measure the concordance between *ica* genes, CRA positivity and TCP positivity. *P*-value was two-tailed and was considered significant at a level of ≤0.05. The descriptive statistic was used to calculate mean values and standard deviation. Data were analyzed by use of STATISTICA v 9.0 (StatSoft Inc., Tulsa, OK, USA) on the Windows platform.

## 3. Results and Discussion

Microbiological samples from hospital environments were collected between November 2011 and May 2012. Samples were collected from air and flat surfaces of the both surgical operating theater (50 samples) and general surgery hospital ward (20 samples), as described in Experimental section. In the operating surgery theater samples were collected from: floor, sterile lock, corridor, cleaning room, preparation room, sterile materials room, post-surgery care room. In the general surgery ward samples were collected from: patients’ facilities, day-operating surgery, baths, nursery office. From each of the aforementioned areas samples were collected twice a day, in the morning and in the afternoon. In the surgical operating theater 83 (35.8%) out of total 236 isolated strains were CoNS while in the general surgery ward that percentage was lower—39 (22.5%) CoNS strains out of a total of 173 strains isolated. The species distribution of the 122 analyzed CoNS strains isolated from hospital environment was as follows: *S. epidermidis*—32 strains, *S. haemolyticus*—31 strains, *S. capitis subsp. capitis*—21 strains, *S. hominis*—11 strains, *S. cohnii subsp. cohnii*—9 strains, *S. saprophyticus*—5 strains, *S. warneri* and *S. kloosii*—4 strains, *S. cohnii subsp. urealyticum*—2 strains and *S. lugdunensis*, *S. hyicus,* and *S. chromogenes*—1 strain each. Isolated CoNS strains demonstrated various susceptibility to tested chemotherapeutics (Table 1).

**Table 1:** The number/percentage of CoNS strains susceptible to selected chemotherapeutics.

Strain (No. of Strains)	^a^ FOX	^a^ E	^a^ DA	^a^ T	^a^ C	^a^ CIP	^a^ CN	^a^ RIF	^a^ LNZ	^a^ SXT
*S. epidermidis* (32)	25/78.1	18/56.3	26/81.3	25/78.1	32/100	32/100	31/96.9	32/100	31/96.9	23/71.9
*S. haemolyticus* (31)	26/83.9	14/45.2	19/61.3	26/83.9	25/80.6	27/87.1	28/90.3	31/100	31/100	18/58.1
*S. capitis subsp. capitis* (21)	17/81	9/42.9	15/71.4	19/90.5	21/100	10/47.6	10/41.6	19/90.5	21/100	17/81
*S. hominis* (11)	10/90.9	4/36.4	9/81.8	6/54.5	9/81.8	11/100	11/100	11/100	11/100	8/72.7
*S. cohnii subsp. cohnii* (9)	7/77.8	5/55.6	5/55.6	5/55.6	9/100	9/100	9/100	9/100	9/100	5/55.6
*S. saprophyticus* (5)	5/100	1/20	4/80	5/100	5/100	5/100	5/100	5/100	5/100	3/60
*S. kloosii* (4)	4/100	2/50	3/75	1/25	3/75	4/100	4/100	4/100	4/100	4/100
*S. warneri* (4)	3/75	1/25	1/25	3/75	3/75	4/100	4/100	4/100	4/100	3/75
*S. cohnii subsp. urealyticum* (2)	2/100	0/0	1/50	1/50	2/100	2/100	2/100	2/100	2/100	1/50
*S. lugdunensis* (1)	1/100	1/100	1/100	1/100	1/100	1/100	1/100	1/100	1/100	1/100
*S. hyicus* (1)	1/100	1/100	1/100	1/100	1/100	1/100	1/100	1/100	1/100	0/0
*S. chromogenes* (1)	1/100	0/0	1/100	0/0	1/100	1/100	1/100	1/100	1/100	1/100
Total (122)/%	10/83.6	56/45.9	86/70.5	93/76.2	112/91.8	107/87.7	107/87.7	120/98.4	121/99.2	84/68.9

^a^ FOX—cefoxitin, E—erythromycin, DA—clindamycin, T—tetracycline, C—chloramphenicol, CIP—ciprofloxacin, CN—gentamicin, RIF—rifampicin, LIN—linezolid and SXT trimethoprim/ sulphamethoxazole.

**Figure 1:**
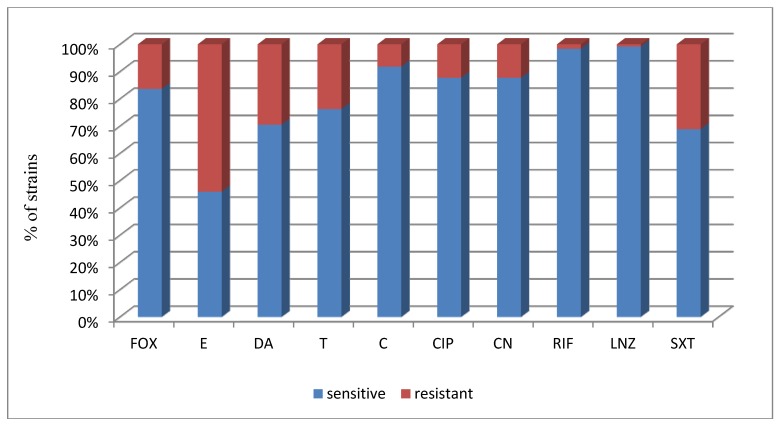
The proportion of susceptible and resistant CoNS strains to the tested chemotherapeutics.

CoNS strains isolated from hospital environment were highly susceptible to linezolid (99.2% sensitive strains) rifampicin and chloramphenicol (98.4% and 91.85% sensitive strains respectively) while erythromycin (45.9% sensitive strains), clindamycin (70.5% sensitive strains) and trimethoprim in combination with sulphamethoxazole (68.9%) showed low antistaphylococcal activity (Figure 1).

FOX—cefoxitin, E—erythromycin, DA—clindamycin, T—tetracycline, C—chloramphenicol, CIP—ciprofloxacin, CN—gentamicin, RIF—rifampicin, LIN—linezolid and SXT trimethoprim/ sulphamethoxazole.

**Figure 2:**
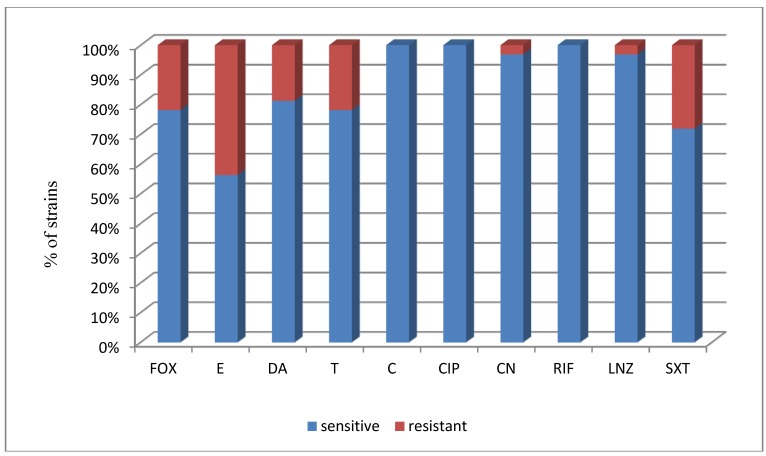
The proportion of susceptible and resistant *S. epidermidis* strains to the tested chemotherapeutics.

What is more, the susceptibility profile for all CoNS strains was very similar for *S. epidermidis* strains alone (Figure 2). *S. epidermidis* strains were highly susceptible to chloramphenicol, rifampicine and ciprofloxacine (100% strains), with lesser susceptibility to erythromycin (56.3% stains) and trimetoprime in combination sulphamethoxazole—71.9% isolated and tested strains.

FOX—cefoxitin, E—erythromycin, DA—clindamycin, T—tetracycline, C—chloramphenicol, CIP—ciprofloxacin, CN—gentamicin, RIF—rifampicin, LIN—linezolid and SXT trimethoprim/ sulphamethoxazole.

Only nine (7.4%) out of 122 tested strains were considered as biofilm-formers by CRA method and what is more, not a single *S. epidermidis* strain was among them.

The biofilm production analysis by TCP method showed 12 (9.8%) biofilm-forming strains (A_490_ > 0.17), and 13 (10.7 %) strains with intermediate biofilm forming ability with A_490_ ranging from 0.11 to 0.16 (Table 2). Among 32 analyzed *S. epidermidis* strains, 12 (37.5%) were assessed as a biofilm formers by this method.

**Figure 3:**
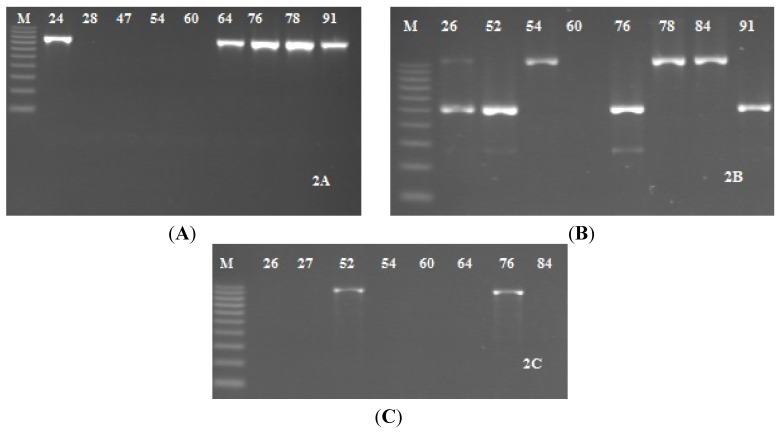
Detection of operon *icaADBC* genes in *Staphylococcus epidermidis* strains. (**A**) PCR results with primer for *icaA*, positive probe—the presence of the 814-bp product; (**B**) PCR results with primer for *icaB* and *icaD*, positive probe—the presence of the 526-bp product to *icaB* and 371-bp product to *icaD*; and (**C**) PCR results with primer for *icaC* genes, positive probe—the presence of the 989-bp product. Line 1—molecular size maker 100-1000-bp, line 2-9 different *S. epidermidis* strains.

Genetic analysis of 32 *S. epidermidis* strains showed the presence of *icaADBC* operon genes in 15 (46.9%) isolates (Figure 3A–C). *IcaA* and *icaD* were present in 34.4% and 28.1% of strains respectively and *icaC* gene in 37.5% of strains (Figure 3A, C). The lowest frequency (21.9%) showed *icaB* gene (Figure 3B). The presence of all *icaADBC* operon genes was found in five (15.6%) *S. epidermidis* strains, while in four (12.5%) isolates we found coexistence of *icaA/icaC*. The frequencies of *icaB/icaD*, *icaA/icaD/icaB*, and *icaA/icaB/icaC* genotypes were on the same level (3.1%) 

**Table 2:** Assessment of biofilm formation ability of CoNS strains determined by CRA and TCP methods.

Strain (No. of strains)	No (%) of Positive Strains, CRA Method	No (%) of Positive Strains, TCP Method OD > 0.17,—Strong Biofilm Production	No (%) of Positive Strains, TCP Method 0.11 > OD >0.16—Weak Biofilm Production
*S. epidermidis* (32)	0	9 (28.1)	3 (9.4)
*S. haemolyticus* (31)	1 (3.2)	1 (3.2)	3 (3.2)
*S. capitis subsp. capitis* (21)	3 (14.3)	0	1 (4.8)
*S. hominis* (11)	1 (9.1)	0	2 (18.2)
*S. cohnii subsp. cohnii* (9)	2 (22.2)	0	0
*S. saprophyticus* (5)	0	0	0
*S. kloosii* (4)	1 (25)	1 (25)	1 (25)
*S. warneri* (4)	0	0	1 (25)
*S. cohnii subsp. urealyticum* (2)	0	1 (50)	1 (50)
*S. lugdunensis* (1)	0	0	0
*S. hyicus* (1)	1 (100)	0	0
*S. chromogenes* (1)	0	0	1 (100)
Total: (122)	9 (7.4)	12 (9.8)	13 (10.7)

Among the 5 *S. epidermidis* strains with all *icaADBC* operon genes, only three isolates showed biofilm formation ability (A_490_ = 0.66 ± 0.25). Among four *icaC/icaA* positive *S. epidermidis* strains, three showed phenotypic ability to biofilm formation (A_490_ = 0.54 ± 0.2). All *S. epidermidis* strains with *ica* genes yielded negative results for biofilm production assessed by the CRA method while 12 strains were considered as biofilm positive in by the TCP method.

There were no significant differences in antimicrobial susceptibility between biofilm forming and non-biofilm strains, neither in the magnitude nor in the resistance pattern (Table 3). This observation suggest that diminished susceptibility to antibiotics of biofilm-forming strains, described previously by some authors [5,6,22] may be due to impaired penetration of the drug across the biofilm rather than to any other biochemical or genetic mechanisms.

**Table 3:** *S. epidermidis* strains susceptibility to chemotherapeutics with correlation to phenotypic biofilm production ability.

	^a ^FOX	^a ^E	^a ^DA	^a ^T	^a ^C	^a ^CIP	^a ^CN	^a ^RIF	^a ^LNZ	^a ^SXT
***S. epidermidis* (32)**										
**No. of susceptible strains**	25	18	26	25	32	32	31	32	31	23
**% of susceptible strains**	78.1%	56.3%	81.3%	78.1%	100%	100%	96.9%	100%	96.9%	71.9%
**biofilm strains (12)**										
**No. of susceptible strains**	9	6	9	9	12	12	11	12	11	10
**% of susceptible strains**	75%	50%	75%	75%	100%	100%	91.7%	100%	91.7%	83.3%
**non biofilm strains (20)**										
**No. of susceptible strains**	16	12	17	16	20	20	20	20	20	13
**% of susceptible strains**	80%	60%	85%	80%	100%	100%	100%	100%	100%	65%
**^b ^*P***	1.000	0.718	0.647	0.535	^c ^NA	NA	0.375	NA	0.375	0.205

^a^ FOX—cefoxitin, E—erythromycin, DA—clindamycin, T—tetracycline, C—chloramphenicol, CIP—ciprofloxacin, CN—gentamicin, RIF—rifampicin, LIN—linezolid and SXT trimethoprim/ sulphamethoxazole ^b^ *p*: the two-sided Fisher’s exact test for biofilm forming strains *vs.* non biofilm forming strains; all presented *P* values are two-sided, *p* ≤ 0.05 was considered as statistically significant. ^c^ NA—not applicable.

Biofilm formation is a relatively common phenomenon among many microorganisms. The balance between biofilm-type and planktonic-type growth is influenced by a vast variety of regulatory mechanisms. To date many factors which exert significant influence on biofilm formation have been identified. Environmental factors such as oxygen and iron ions availability and high osmotic pressure can influence extracellular matrix biosynthesis, thus the biofilm formation as a whole [31].

In our study we have analyzed the prevalence of coagulase-negative staphylococci with biofilm formation ability isolated from hospital environment. We applied three widely used methods, such as: the growth rate on the CRA, evaluation of the biofilm formation ability by TCP method and PCR-based detection of *icaADBC* operon genes associated with biofilm formation.

We showed that only 7.4% of all analyzed CoNS strains yielded positive reactions in the CRA method. Among *S. epidermidis* none of strains showed the ability to form biofilms when assessed by this method, while the presence of *icaABCD* operon genes associated with the biofilm formation was found in 15 (46.9%) *S. epidermidis* isolates. The biofilm production by hospital environment CoNS strains estimated by TCP method showed that 20.5% of isolates were able to produce biofilm. Our data strongly suggest that the use of TCP method together with the PCR-based techniques should be used as a gold standard for the evaluation of biofilm formation ability by CoNS strains isolated from hospital environments. This methodological problem was also addressed by Mathur *et al.* [32] who compared three phenotypic methods used for determination of biofilm formation. The authors evaluated 152 clinical isolates of *Staphylococcus* spp*.* by CRA, TCP and tube (TM) methods. Their results showed that 97.1% of strains were assessed as biofilm producers with the use of TCP method, 73.6% by TM, and only 6.8% in the case of CRA method, thus the authors concluded that the most sensitive method for biofilm formation analysis was the TCP method. The low effectiveness of the CRA method in evaluation of biofilm production, was also shown by de Silva *et al*. [29]. In these studies they analyzed strains of coagulase-negative staphylococci isolated from blood and skin of neonatal patients and healthy newborns. Among the 180 strains, 122 (68%) was *S. epidermidis,* *S. capitis* (29), *S. haemolyticus* (11), *S. hominis* (9), *S. warneri* (8), and *S. auricularis* (1). The majority of analyzed strains have not demonstrated the phenotypic ability of biofilm formation in TCP and CRA methods, with the exception of *S. hominis* isolates, where four out of nine strains were positive in the CRA method. The authors concluded that the quantification of biofilm formation by absorbance measurement on titrate plates is significantly higher than using the CRA method.

Oliveira and Cucha confirmed the relatively high efficiency of the microplate method [21]. Analysis of 100 CoNS strains isolated from clinical specimens obtained from newborns and tested with the method described by Christensen *et al.*, showed that 46% of isolated were characterized by strong phenotypic capacity for biofilm production, while 35% of them were weak biofilm producers. The most frequently isolated strain from clinical specimens identified in this study was *S. epidermidis* (81%) with *S. cohnii, S. saprophyticus, S. warneri, S. haemolyticus, S. xylosus, S. capitis,* and *S. lugdunensis* composing the remaining 19%.

Our data have shown that among coagulase-negative staphylococci from hospital environments, the most frequent were *S. epidermidis* (26.2%), *S. haemolyticus* (25.4%), *S. capitis* (17.2%), which indicates that these strains may be transferred from the hospital environment to the patient and may be responsible for hospital-acquired infections. A study carried out in the hospital environment by Wojtyczka *et al.* [33] showed that among 85 isolated strains of *Staphylococcus* spp. most common species of CoNS were *S. epidermidis* (17.7%), *S. hominis* (15.3%), *S. cohnii* (14.1%) and S. *haemolyticus* (12.9).

It is worth mentioning that the results presented here indicate a strong predominance of *S. epidermidis* isolates over other CoNS strains isolated from the clinical samples wereas such a phenomenon is less notable among CoNS strains isolated from the general hospital environment. This observation reinforces the notion that biofilm-forming *S. epidermidis* strains colonizing the hospital environment are responsible for an increased risk of nosocomial infections.

It is believed that the presence of *icaABCD* operon genes in the staphylococcal genome s associated with biofilm formation ability. Fredheim *et al.* [34], suggested a strong similarity between *ica* operons in different species of staphylococci. Phylogenetic analysis showed a substantial likeness of *ica* operon primary structure in such species as *S. haemolyticus* and *S. epidermidis.* Oliveira and Cucha’s study [21] on the prevalence of *icaA, icaC* and *icaD* genes in CoNS strains showed that the *icaA* and *icaD* genes were present in 40% of isolates, and *icaA, icaC* and*icaD* in 42% of strains. In our study only 18% of analyzed strains lacked all *icaABCD* genes. In studies carried out by Arciola *et al.* [35] 101 *S. aureus* and *S. epidermidis* clinical isolates were checked for the presence of *icaA* and *icaD* genes. Sixty strains of *S. epidermidis* and 23 strains of *S.* *aureus* were isolated from infections associated with the implementation of vascular catheter and 10 strains of *S. epidermidis* were isolated from the skin and mucous membranes of healthy subjects. The presence of both genes (*icaA* and *icaD*) were confirmed in 48.5% (33) of *S. epidermidis* clinical strains. The remaining 35 strains of *S. epidermidis* and 10 strains isolated from the skin and mucous membranes, lacked *ica* operon genes. The results coincided with the phenotypic assessment of biofilm formation by CRA method. On the other hand, many experiments have shown the low usefulness of the CRA method for biofilm production analysis and this was also confirmed in the presented work where such a coincidence was not observed. Phenotypic methods for biofilm formation detection are among the easy and cheap techniques available for routine laboratory use, but may cause some difficulties in result interpretation since they can be influenced by variations in medium composition and cultivation conditions and are prone to subjective errors [36].

Our analysis of CoNS strains isolated from hospital environment showed the coexistence of all *icaADBC* operon genes in eight (6.6%) of all isolates and among them in five (15.63) *S. epidermidis* strains. 

Only two strains *S. epidermidis* and *S. cohnii subsp. urealyticum* had the ability to produce biofilm when assessed by the TPC method but lacked *icaADBC* operon genes, which is in accordance with an observation presented by Qin *et al*. [37]. The authors showed that the *aap* and *bhp* genes may be involved in an alternative PIA-independent mechanism of biofilm formation, thus indicating that the absence of *icaABCD* operon genes does not exclude biofilm formation. Assessing the ability of biofilm production by two groups of *S. epidermidis* strains isolated from nosocomial infections and skin of healthy subjects, Eftekhar and Mirmohamadi [38] showed that the prevalence of biofilm-forming strains in the TPC method was at the 52% and 56% level for the analyzed groups, respectively. The application of molecular techniques with single pair of primers for the *icaA, icaB* and *icaD* yielded positive results in 30% *S. epidermidis* isolates from nosocomial infections and in 8% of the isolates from the skin of healthy volunteers.

The increased frequency of strains resistant to many antibiotics and chemotherapeutics is responsible for a substantial number of infections in hospital environments. The resident hospital microflora is relatively dynamic and susceptibility and resistance patterns of microorganisms isolated from clinical environmental samples can vary significantly [39]. 

Our study demonstrated that CoNS strains isolated from hospital environment showed high susceptibility towards the majority of tested chemotherapeutics, and susceptibility to resistance patterns only slightly varied among investigated CoNS species.

Many studies showed that resistance of CoNS to selected antibiotics can vary among strains within a broad range [39,40,41,42,43,44,45,46]. It has been shown that proportion of susceptible to resistant strains to erythromycin varied from 29.5% to 73.4%, to clindamycin from 34% to 70.3% strains, to ciprofloxacin from 6.4% to 59.4%, and to gentamicin from 15% to 42.3% strains. The proportion of resistant Staphylococci strains to trimethoprim/sulphamethoxazole varied from 24% to 40.7%, to chloramphenicol from 0% to 60.9%, to tetracycline from 13.1% to 51.9%, and to rifampicin from 7% to 39.1% [39,40,41,42]. Our study confirmed high resistance of CoNS to lincosamides and macrolides. On the contrary to data presented by others [43,44], ciprofloxacin was substantially more effective towards CoNS and, what is worth mentioning, all analyzed *S. epidermidis* strains were susceptible to that drug. What is more, analyzed *S. epidermidis* strains were highly susceptible to rifampicin. This observation is in accordance with previous findings describing rifampicin as a potent agent against biofilm forming CoNS strains [45,46] thus it may be used in therapies against infections associated with biofilm forming *S. epidermidis* strains.

## 4. Conclusions

Our results have confirmed previous data presented by other authors that the molecular presence of *icaADBC* operon genes in the bacterial genome is associated with the ability to form biofilms, but the absence of these genes does not preclude this phenomenon phenotypically. Therefore, it seems appropriate to use both genotypic and phenotypic methods to improve the identification of the ability to produce biofilms by CoNS strains isolated from the hospital environment. Despite the fact that the analyzed CoNS strains were in the majority susceptible to the tested chemotherapeutics, a substantial contribution of biofilm-forming strains among them may cause problems in chemotherapy of hospital infections, particularly in the dose assessment. In this light, it is obvious that the information about bacterial species prevailing in hospital environment, their susceptibility and resistance patterns and on their biofilm formation ability is vital for both health care providers for the implementation of hospital infection prevention and control plans and for physicians in building up adequate antibacterial therapies.

## References

[1] European Centre for Disease Prevention and Control (ECDC) (2007). Annual Epidemiological Report on Communicable Diseases in Europe.

[2] Lis D.O., Pacha J.Z., Idzik D. (2009). Methicillin resistance of airbiorne coagulase-negative staphylococci in homes of persons having contact with a hospital environment. Am. J. Infect. Control.

[3] Bryers J.D. (2008). Medical biofilms. Biotechnol. Bioeng..

[4] Kampf G., Löffler H., Gastmeier P. (2009). Hand hygiene for the prevention of nosocomial infections. Dtsch. Ärztebl. Int..

[5] Høiby N., Bjarnsholta T., Givskovb M., Molinc S., Ciofub O. (2010). Antibiotic resistance of bacterial biofilms. Int. J. Antimicrob. Agents.

[6] Nieshimura S., Tsurumoto T., Yonekura A., Adachi K., Shindo H. (2006). Antimicrobial susceptibility of* Staphylococcus aureus* and *Staphylococcus epidermidis* biofilms isolated from infected total hip arthroplasty case. J. Orthoped. Sci..

[7] Presterl E., Suchomel M., Eder M., Reichmann S., Lassnigg A., Graninger W., Rotter M. (2007). Effects of alcohols, povidone-iodine and hydrogen peroxide on biofilms of *Staphylococcus epidermidis*. J. Antimicrob. Chemother..

[8] Costerton J.W., Stewart P.S., Greenberg E.P. (1999). Bacterial biofilms: A common cause of persistent infections. Science.

[9] Izano E., Amarante M., Kher W., Kaplan J. (2008). Differential roles of poly-*N*-acetylglucosamine surface polysaccharide and extracellular DNA in *Staphylococcus aureus* and *Staphylococcus epidermidis* biofilms. Appl. Environ. Microbiol..

[10] Otto M. (2009). *Staphylococcus epidermidis—*the “accidental” pathogen. Nat. Rev. Microbiol..

[11] Mack D., Fischer W., Krokotsch A., Leopold K., Hartmann R., Egge H., Laufs R. (1996). The intercellular adhesin involved in biofilm accumulation *Staphylococcus epidermidis* linear beta-1,6-linked glucosaminoglycan: purification and structural analysis. J. Bacteriol..

[12] Darby C., Hsu J.W., Ghori N., Falkow S. (2002). *Caenorhabditis. elegans*: Plague bacteria biofilm blocks food intake. Nature.

[13] Kaplan J.B., Velliyagounder K., Ragunath Ch., Rode H., Mack D., Knobloch J.K., Ramasbbu N. (2004). Genes involved in the synthesis and degradation of matrix polysaccharide in *Actinobacillus. actinomycetemcomitans* and *Actinobacillus. pleuropneumoniae* biofilms. J. Bacteriol..

[14] Wang X., Preston J.F.I., Romeo T. (2004). The *pgaABCD* locus of *Escherichia coli* promotes the synthesis of a polysaccharide adhesin required for biofilm formation. J. Bacteriol..

[15] Vuong C., Kocianova S., Voyich J.M., Yao Y., Fishcer E.R., DeLeo F.R., Otto M. (2004). A crucial role for exopolysaccharide modification in bacterial biofilm formation, immune evasion, and virulence. J. Biol. Chem..

[16] Gerke C., Kraft A., Sussmuth R., Schweitzer O., Götz F. (1998). Characterization of the N-acetylglucosaminyltransferase activity involved in the biosynthesis of the *Staphylococcus epidermidis* polysaccharide intercellular adhesion. J. Biol. Chem..

[17] Rupp M.E., Fey P.D., Heilmann C., Götz F. (2001). Characterization of the importance of *Staphylococcus epidermidis* autolysin and polysaccharide intercellular adhesin in the pathogenesis of intravascular catheter-associated infection in a rat model. J. Infect. Dis..

[18] Fluckiger U., Ulrich M., Steiuhuber A., Döring G., Mack D., Landmann R., Goerke Ch., Wolz Ch. (2005). Biofilm formation, *icaADBC* transcription, and polysaccharide intercellular adhesin synthesis by staphylococci in a device-related infection model. Infect. Immun..

[19] Costa F.S., Miceli M.H., Anaissie E.J. (2004). Mucosa or skin as source of coagulase-negative staphylococcal bacteraemia. Lancet Infect. Dis..

[20] Klingenberg C., Aarag E., Ronnestad A., Sollid J.E., Abrahamsen M.D., Kjeldsen G., Flaegstad T. (2005). Coagulase-negative staphylococcal sepsis in neonates—Association between antibiotic resistance, biofilm formation and the host inflammatory response. Pediatr. Infect. Dis. J..

[21] Oliveira A., Cunha M.L.R.S. (2010). Comparison of methods for the detection of biofilm production in coagulase-negative staphylococci. BMC Res. Notes.

[22] O’Gara J., Humphreys H. (2001). *Staphylococcus epidermidis* biofilms: Importance and implications. J. Med. Microbiol..

[23] Cogen A.L., Nizet V., Gallo R.L. (2008). Skin microbiota: A source of disease or defence?. Brit. J. Dermatol..

[24] Hart J.B., French M.L.V., Eitzen H.E., Ritter M.A. (1973). Rodac plate-holding device for sampling surfaces during surgery. Appl. Microbiol..

[25] Lemmen S.W., Häfner H., Zolldann D., Amedick G., Lutticken R. (2001). Comparison of two sampling methods for the detection of Gram-positive and Gram-negative bacteria in the environment: Moistened swabs *versus* Rodac plates. Int. J. Hyg. Environ. Health.

[26] Freeman D.J., Falkiner F.R., Keane C.T. (1989). New method for detecting slime production by coagulase negative staphylococci. J. Clin. Pathol..

[27] Christensen G.D., Simpson W.A., Younger J.J., Baddour L.M., Barrett F.F., Melton D.M., Beachey E.H. (1985). Adherence of coagulase-negative staphylococci to plastic tissue culture plates: A quantitative model for the adherence of staphylococci to medical devices. J. Clin. Microb..

[28] Ziebuhr W., Krimmer V., Rachid S., Lößner I., Götz F., Hacker J. (1999). A novel mechanism of phase variation of virulence in *Staphylococcus epidermidis*: Evidence for control of the polysaccharide intercellular adhesin synthesis by alternating insertion and excision of the insertion sequence element IS256. Mol. Microbiol..

[29] De Silva G.D.I., Kantzanou M., Justice A., Massey R.C., Wilkinson A.R., Day N.P.J., Peacock S.J. (2002). The *ica* operon and biofilm production in coagulase-negative staphylococci associated with carriage and disease in a neonatal intensive care unit. J. Clin. Microbiol..

[30] European Committee for Antimicrobial Susceptibility Testing (EUCAST) of the European Society of Clinical Microbiology and Infectious Diseases (ESCMID) (2000). Terminology relating to methods for the determination of susceptibility of bacteria to antimicrobial agents. EUCAST definitive document E. Def 1.2. Clin. Microbiol. Infect..

[31] Otto M. (2008). Staphylococcal biofilms. Curr. Top. Microbiol. Immunol..

[32] Mathur T., Singhal S., Khan S., Upadhyay D.J., Fatma T., Rattan A. (2006). Detection of biofilm formation among the clinical isolates of staphylococci: An evaluation of three different screening methods. Ind. J. Med. Microbiol..

[33] Wojtyczka R.D., Krakowian D., Marek Ł., Skiba D., Kudelski A., Jasik K., Pacha J. (2011). Analysis of the polymorphism of *Staphylococcus* strains isolated from a hospital environment. Afr. J. Microbiol. Res..

[34] Fredheim E.G.A., Klingenberg C., Rohde H., Frankenberger S., Gaustad P., Flægstad T., Solid J.E. (2009). Biofilm Formation by *Staphylococcus haemolyticus*. J. Clin. Microbiol..

[35] Arciola C.R., Baldassarri L., Montanaro L. (2001). Presence of *icaA* and *icaD* genes and slime production in a collection of staphylococcal strains from catheter-associated infections. J. Clin. Microbiol..

[36] Růžička F., Holá V., Vota M., Tejkalová R., Horvát R., Heroldová M., Woznicowá V. (2004). Biofilm detection and the clinical significance of *Staphylococcus epidermidis* isolates. Folia Microbiol..

[37] Qin Z., Yang X., Yang L., Jiang J., Ou Y., Molin S., Qu D. (2007). Formation and properties of *in vitro* biofilms of ica-negative *Staphylococcus epidermidis* clinical isolates. J. Med. Microbiol..

[38] Eftekhar F., Mirmohamadi Z. (2009). Evaluation of biofilm production by *Staphylococcus epidermidis* isolates from nosocomial infections and skin of healthy volunteers. Int. J. Med. Med. Sci..

[39] Kochman M. (2005). Susceptibility of the bacteria isolated from samples of clinical material in Poland in 1998 to selected chemotherapeutics and antibiotics. The analysis of the questionnaire findings. I. Susceptibility of staphylococci. Przegl. Epidemiol..

[40] Michnowska-Swincow E., Szychlińska I. (2001). Drug resistance of methicillin resistant coagulase-negative staphylococci (MRCNS) isolated from hospital environment. Diag. Labor..

[41] Piette A., Verschraegen G. (2009). Role of coagulase-negative staphylococci in human disease. Vet. Microbiol..

[42] Tunger O., Ozbakkaloglu B., Aksoy H. (2001). Trends in antimicrobial resistant staphylococci in an university hospital over a 6-year period. Int. J. Antimicrob. Agents.

[43] Olivares M.J., Orozco R.H., Garrido S.R., Rodríguez-Vidigal F.F., Tomé A.V., Marcos M.R. (2011). Activity of vancomycin, ciprofloxacin, daptomycin and linezolid against coagulasenegative staphylococci bacteremia. Rev. Esp. Quimioter..

[44] Sarathbabu R., Rajkumari N., RamaNi V. (2013). Characterization of Coagulase negative Staphylococci isolated from urine, pus, sputum and blood samples. Int. J. Pharma. Sci. Inv..

[45] Zheng Z., Stewart P.S. (2002). Penetration of Rifampin through *Staphylococcus epidermidis* Biofilms. Antimicrob. Agents Chemother..

[46] Leite B., Gomes F., Teixeira P., Souza C., Pizzolitto E., Oliveira R. (2011). *In vitro* activity of daptomycin, linezolid and rifampicin on *Staphylococcus epidermidis* biofilms. Curr. Microbiol..

